# Comparative analysis of anchorage strength and histomorphometric changes after implantation of miniscrews in adults and adolescents: an experimental study in Beagles

**DOI:** 10.1186/s12903-023-03318-y

**Published:** 2023-09-05

**Authors:** Yi Zhao, TingTing Jia, Zhiqiang Wang

**Affiliations:** Department of Orthodontics, Shandong Provincial Hospital Affiliated to Shandong First Medical University, 324 Jingwu Road, Jinan, Shandong China

**Keywords:** Orthodontic miniscrews, Anchorage strength, Histomorphometry, Adult, Adolescent

## Abstract

**Objectives:**

This study aimed to explore the differences in anchorage strength and histomorphometric changes in orthodontic miniscrews between adult and adolescent beagles.

**Material and method:**

Six adult beagles and six young beagles were used as experimental subjects, and eight miniscrews were symmetrically placed in the posterior mandible of each dog. Measurement of the displacement (mm) of two adjacent miniscrews after load application was performed to compare the anchorage strength between the adult and adolescent groups. Three intravital bone fluorochromes (oxytetracycline, calcein green, xylenol orange) were administered postoperatively to mark the active bone-forming surface. Subsequently, the mineral apposition rate and bone-implant contact ratio were measured for dynamic and static histomorphometry. Finally, the expression levels of the RANKL/OPG ratio were evaluated by immunohistochemistry.

**Results:**

The average displacement of miniscrews in the adult group was significantly less than that in the adolescent group after load application. For histomorphometry analysis, the mineral exposure rate in the adolescent group was higher than that in the adult group with or without force application. In addition, more fractures and new bone formation but deceased bone-implant contact ratios were observed in the adolescent group than in the adult group. The ratio of RANKL/OPG expression increased more in the adolescent group than in the adult group.

**Conclusion:**

Miniscrews do not remain in the same position as skeletal anchors, and the amount of displacement was higher in adolescent group than that in adult group, reflecting the weaker anchorage strength of miniscrews in adolescents due to the higher bone turnover rate and active bone remodelling. Therefore, it is feasible to apply orthodontic loading to the miniscrews in adult patients earlier, even immediately, but it is recommended to wait a period for the adolescents.

**Supplementary Information:**

The online version contains supplementary material available at 10.1186/s12903-023-03318-y.

## Introduction

Anchorage, defined as resistance to undesired tooth movement, is the key management strategy during orthodontic treatment. To enhance anchorage, many mechanical apparatuses have been designed and applied in orthodontics. Among these, orthodontic miniscrews are preferred because of their small size, location flexibility, low cost and convenient operation. Above all, miniscrews provide the stronger skeletal anchorage without the cooperation of patients compared with conventional anchorage reinforcement [1]. Therefore, miniscrews are widely used in various malocclusions, such as maxillary protrusion, mandibular retrusion, anterior deep overjet, and excessive tooth display [2–4]. During treatment, miniscrews must keep stable in bone; thus, many researchers have worked to improve osseointegration by imparting biocompatibility to the surface, adequate statistical analysis of anatomical structures, optimization of mechanical properties, enhancement of corrosion resistance and so on [5, 6].

Although the efficacy and safety of miniscrews have been systematically demonstrated, the failure and looseness of miniscrews are still frequently encountered [7–9]. Most previous studies reported that the success rate of miniscrews varies between approximately 70% and 90%, which could be influenced by various host and objective factors including bone quality, miniscrew design, placement technique and loading conditions (period, magnitude, direction, etc.) [10–12]. One of the important host factors was the age of patients. A recent study that evaluated 889 miniscrews in 347 patients showed that younger people with miniscrews inserted in the retromaxillary or retromandibular regions had a higher progressive susceptibility to failure [13]. Several studies also found that the success rate of miniscrews is lower among patients younger than 20 years [14–16]. However, a recent systematic literature review and meta-analysis identified some controversies surrounding the possible association between miniscrew failure and age [17]. Several studies showed no association between age and failure [18, 19]. Moreover, it has been revealed there was a 5% increase in failure risk for every one-year increase in age among participants older than 30 years by a Cox proportional-hazards model [20].

Although many potential risk factors associated with miniscrews are known, loosening of miniscrews still occurs frequently in clinical orthodontic treatment due to the increasing enormous number of patients [21], and the cause of the different failure rates in adolescents compared with adults is still unclear. Many orthodontists proposed that this difference between adults and growing patients in stability may be related to their difference in bone density and cortical bone thickness [22–24]. However, most studies provide evidence mainly based on clinical indicators or radiographic image analysis [25, 26]. In this study, we attempted to investigate the difference in anchorage strength and histomorphometric changes between adolescents and adults by an experimental in vivo model to provide a theoretical basis for better clinical application of miniscrews.

## Materials and methods

### Animal preparation

**Fig. 1:**
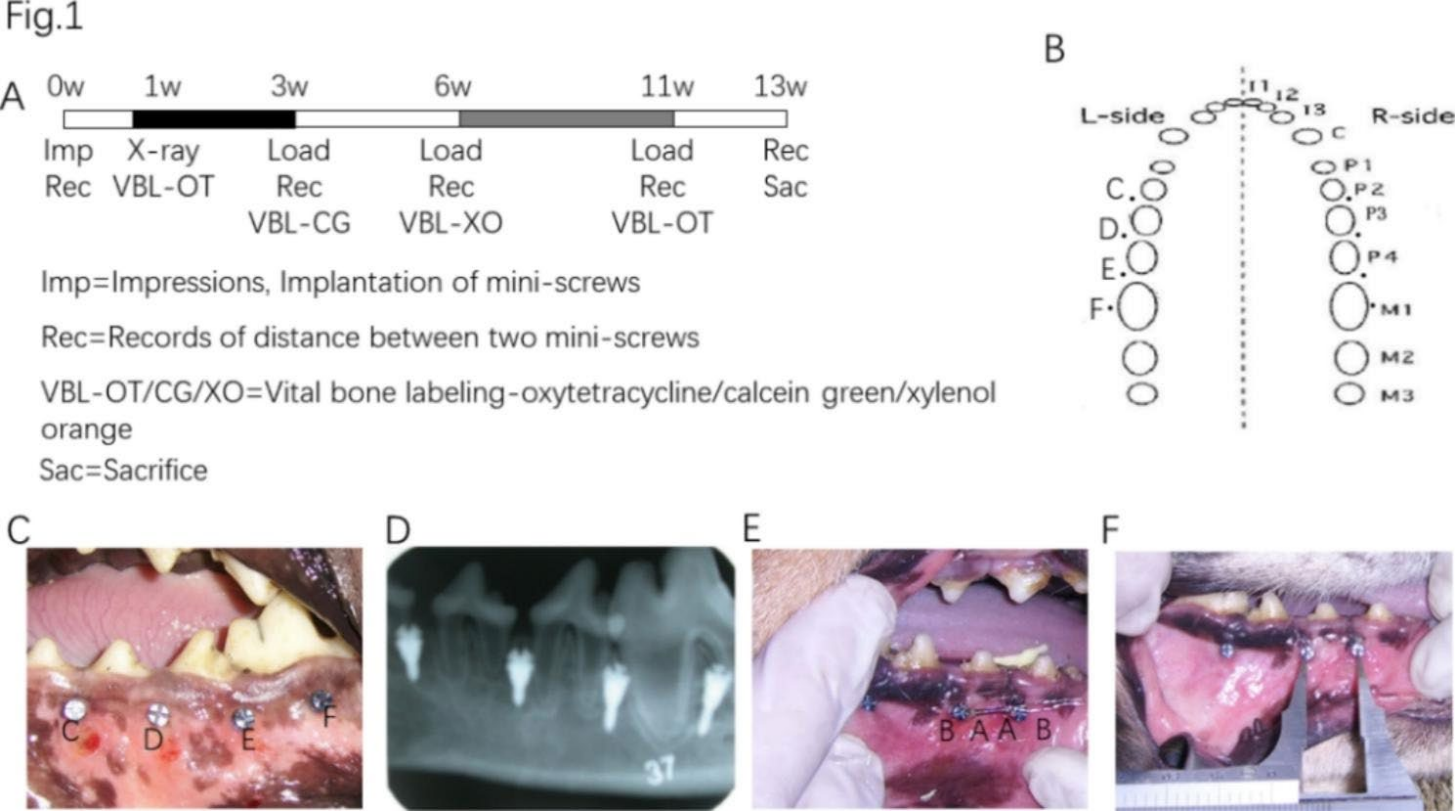
Experimental design and timeline. **A**. Illustration of the experimental timeline and (**B**) the implantation site of miniscrews. **C**. Intraoral photography after the completion of implantation. **D**. Dental X-rays were taken after one week to check the placement. **E**. Force was applied to the interradicular miniscrews **C** and **D** ipsilaterally by a nickel titanium tension spring connecting them; side **A** is the side with the same direction of force applied to the miniscrews, and side **B** is the other side. **F**. Measurement of the distance between miniscrews using a Vernier calliper

A total of twelve male beagles were used in this randomized controlled trial study. Six of them were aged 19–20 months (with eruption of the mandibular third molar, weight 13–14 kg) and were defined as the adult group, and the other six that were aged 6.2–6.8 months (mandibular second and third molars without eruption, weight 8-8.5 kg) were defined as the adolescent group. All animal experiments were approved by the Animal Ethics Committee of Provincial Hospital Affiliated with Shandong University (NO.2,018,091), and all experiments were performed in accordance with the Animal Ethics Procedures and Guidelines of the People’s Republic of China. The animals were observed for 2 weeks to confirm that they were healthy and adapted to the new feeding location before the study. During the entire process, the living environment, food and other external factors of the two groups of animals were ensured to be consistent and stable. The experiments were performed according to a timeline (Fig. 1A).

### Implantation of miniscrews

All procedures were performed under systemic (30 mg/kg 3% sodium pentobarbital) and local infiltration (2% lidocaine with 0.01% epinephrine) anaesthesia. Eight commercially pure titanium miniscrews (diameter 1.6 mm, screw thread length 6.0 mm, Medicon, Germany) were implanted in each animal through the drilling method, four on each side. The miniscrews were scheduled to be placed in the area of premolars and molars in the mandible based on the anatomic features of beagles. Side (left or right) and site (anterior, middle, or posterior) were alternatively assigned for each dog, and the implant sites were the interroots of P2 and P3, P3 and P4, P4 and M1, and proximal and distal M1 (P = premolars; M = molars), which were defined as C, D, E, and F, respectively (Fig. 1B-C). We implanted the miniscrews at suitable sites with an insertion angle ranging from 60° to 70°, and the height to the alveolar bone crest was as consistent as possible. After surgery, 800,000 units of penicillin were injected intramuscularly every day for 7 consecutive days. Dental X-rays were taken after one week to check the placement (Fig. 1D), and all surgical procedures were performed by the same operator. During the subsequent evaluation, the researchers who were in charge of the data analysis process were not aware of the group allocation to ensure the accuracy of the results.

### Evaluation of the anchorage strength of miniscrews

To evaluate the anchorage strength of miniscrews, the distance between miniscrews was measured before and after accepting stretching force. Three weeks after implantation, the forces were applied to the interradicular miniscrews C and D ipsilaterally by a nickel titanium tension spring connecting them (Fig. 1E). All groups were assigned to have constant stretch tension of 150 g bilaterally, and the nickel titanium tension spring was adjusted after reloading to maintain the force at the same level. The distance between them was measured with a Vernier calliper at three time points: 0 w, 3 w, and 10 w. The measuring point was the central point on the surface of the head of the miniscrews with two forces, and the average value was taken 3 times each time by the same operators (Fig. 1F). All loading and measurements were performed by the same operator, and the above procedures and the injection of fluorescent markers were performed under general anaesthesia.

### Intravital bone fluorochrome staining

Four spots after implantation were intravenously injected four times within 11 weeks to mark active bone-forming surfaces: oxytetracycline (20 mg/kg) (Terramicin Long-acting, Zoetis, Italy) was injected 1 week after implantation; calcein green (8 mg/kg) was injected 3 weeks after the operation (Sigma C0875); and xylenol orange (120 mg/kg) (Fluka 33,825) was injected 6 weeks after the operation. At 11 weeks after surgery, oxytetracycline (20 mg/kg) (Terramicin Long-acting, Zoetis, Italy) was injected. The in vivo experimental design is shown in Fig. 1.

### Specimen preparation

Thirteen weeks after implantation, all animals were sedated and anaesthetized as previously described. The carotid artery was perfused with 4% neutral paraformaldehyde, and the femoral artery was exsanguinated. The mandible containing miniscrews was separated and removed, and then the lower margin of the mandible was trimmed to guarantee that the longitudinal axis of the bone was parallel to the direction of the force. The left specimen was fixed using 4% paraformaldehyde for decalcification, and the right specimen was immersed in 75% alcohol for undecalcified bone processing. After dehydration and infiltration, the mandibular specimen was embedded in poly(methyl methacrylate) (Merck, Schuchardt, Hohenbrunn, Germany). Then, each block was sectioned by using a saw microtome (SP 1600; Leica Instruments, Nussloch, Germany), obtaining a series of Sect. 60 μm in thickness. One unstained section of each specimen was used for vital bone fluorescence labelling observation, and three consecutive sections of the central portion of the miniscrew site were also used for static histomorphometric evaluation after being stained with 1% toluidine blue.

### Histologic observation and histomorphometric evaluation

One unstained section for each specimen was placed under a fluorescence microscope (OLYMPUS IX71). Fluorescence images of the tail, middle and neck of the miniscrews were collected, and the mineral apposition rate (MAR, µm/day) was calculated for dynamic histomorphometry with Image Pro-plus software to evaluate the rate of new bone formation. The MAR is the rate of progression of the mineralization front between two consecutive fluorochromes as an index of osteoblast activity. In addition, static histomorphometric evaluations were carried out with Leitz Microvid equipment connected to an IBM XT computer with an image analysis system (Nikon Em120; ACT-1). Image-Pro Plus software was used to perform static bone morphometry at the implant-bone interface. The bone-implant contact ratio (BIC) was calculated by the following formula: BIC (%) = implant surface length in contact with osseous tissue ÷ total surface length of implant × 100%.

### Immunohistochemistry staining

Paraffin-embedded sagittal sections of decalcified mandible tissue were sliced 5 μm thick, deparaffinized in xylene two times and dehydrated with gradient concentrations of ethanol. To block endogenous peroxidase activity, the slides were incubated with 3% hydrogen peroxide for 15 min and blocked with 5% BSA for 30 min at room temperature to prevent nonspecific binding. Then, the slides were incubated with the primary antibodies anti-OPG (1:50, ab73400; Abcam) and anti-RANKL (1:200, ab9957; Abcam) at 4 °C overnight. After being immersed in a secondary antibody for 1 h at room temperature, the sections were washed with PBS (pH 7.2–7.6) three times for 5 min each. Then, 3,3’- diaminobenzidine (DAB) (ZLI-9018; Zhongshan Jinqiao Biotechnology Co.) was used for the colour reaction, which was stopped with distilled water, followed by counterstaining with haematoxylin. Finally, dehydration and sealing were performed with neutral gum. Slides were photographed using a light microscope (Olympus, Tokyo, Japan), and the fractional (%) stained area was calculated using Image-Pro Plus software.

### Statistical analysis

All in vitro experiments were performed at least three times, and the measurements were conducted twice by the same investigator at an interval of 2 weeks. Reliability tests were applied to ensure the stability, internal consistency and equivalence of the main measurement properties. Independent-sample *t* tests were used to compare the differences between the adult group and the adolescent group in the displacement of miniscrews, MAR and BIC. Statistical analysis was carried out with the SPSS 10.0 software package. Differences with probabilities less than 5% (*P* < 0.05) were considered statistically significant.

## Results

### Evaluation of the anchorage strength of miniscrews

All miniscrews were successfully implanted and no loose or failed screws were found in the healing stage. To evaluate the anchorage strength of the miniscrews, the distance between the miniscrews was measured and recorded after accepting mechanical force (Table S1). Then the displacement of the miniscrews was calculated and is shown in Table 1. The observation variables were independent, normally distributed and the variance between the two groups of observation variables was equal. The observations revealed that the average displacement of the adult group (n = 12) was less than that of the adolescent group (n = 12) at the three loading time points, and the difference was statistically significant (P < 0.01). Furthermore, the displacement of the miniscrews in the first 3 weeks was greater than that in the last 7 weeks, which indicated that the displacement of miniscrews mainly occurred in the first 3 weeks.

**Table 1:** Displacement of miniscrews after mechanical force (mm)

		Loading Time	
Group	0–3 w	3–10 w	0–10 w
Adults	0.1800 ± 0.07385	0.1083 ± 0.04896	0.2883 ± 0.11961
Adolescents	0.3925 ± 0.11153	0.2350 ± 0.02355	0.6250 ± 0.15681
*p* value	*p* < 0.01	*p* < 0.01	*p* < 0.01

### Evaluation of bone fluorochrome staining

**Fig. 2:**
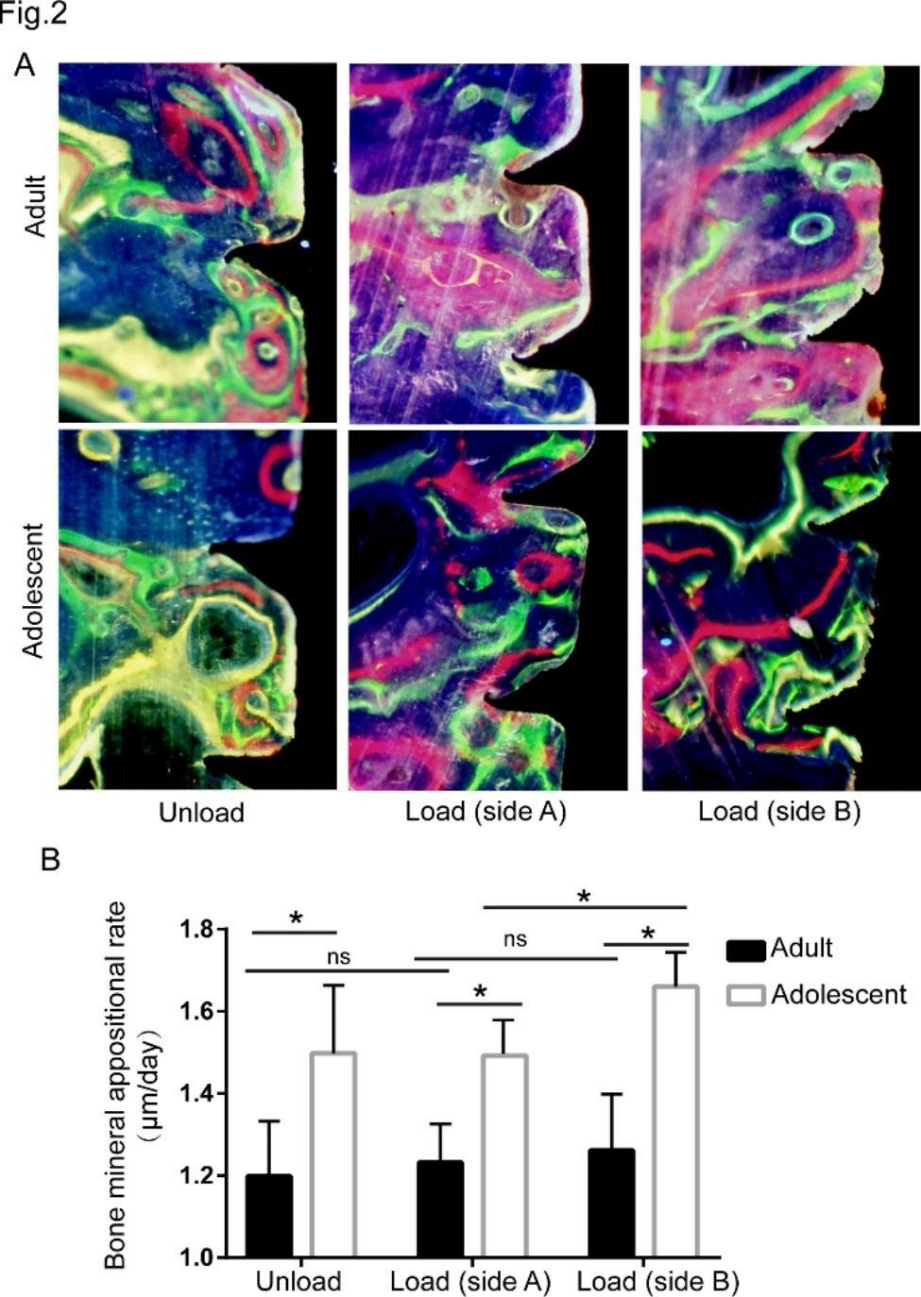
Evaluation of bone fluorochrome staining. **A**. Fluorescence microscopic images with labelling of oxytetracycline, calcein green and xylenol orange. Fluorescent labelling demonstrated the development of newly formed bone. **B**. The bone mineral appositional rate (MAR) was evaluated and compared with the corresponding controls

Fluorescent labelling demonstrated the development of newly formed bone. There were three fluorescent labels in the middle part of the miniscrew (Fig. 2A). In unloading screws, we could see three fluorescent bands: green near the margin, red and yellow outwards. In loading screws, we could see fluorescent expression around the implant, but there were no obvious bands. Moreover, there was more obvious bone deposition on side B than on side A (A was the side with the same direction of force applied to the miniscrew, and side B was the other side). The bone mineral appositional rate (MAR) was determined by measuring the distance between the centres of the two fluorescent bands divided by the time of labelling, which reflected how fast the mineralization front proceeded during bone remodelling. The results showed that the MAR in the adolescent group was higher than that in the adult group with or without spring application (Fig. 2B), indicating that the speed of bone reconstruction around unstrained miniscrews was faster in the adolescent group than in the adult group.

### Observation of the miniscrew-bone interface

**Fig. 3:**
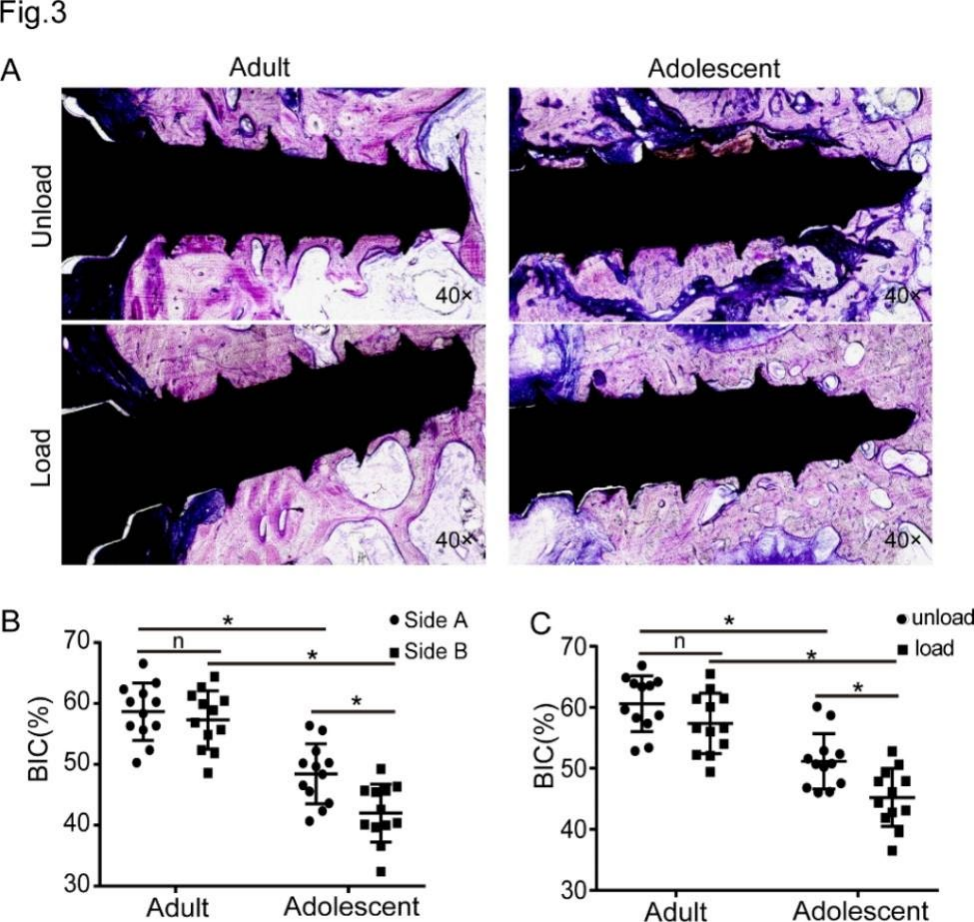
Observation of the miniscrew-bone interface. **A**. Histological images of the miniscrew-bone interface in two groups with or without loading. **B**. The analysis of the BIC compared with corresponding controls

Of the 96 miniscrews enrolled, the BIC percentage ranged from 32.37 to 66.85%. The means and SDs of the BIC values are presented in Table S2. The staining of undecalcified sections (Fig. 3A) revealed that the miniscrews bound well to the bone in the unloading groups, and fractures and new bone formation were observed in the adolescent group when compared with the adult group. Additionally, more osteoids were formed in the space between the screws and bone in the loading groups of adolescents. For quantitative analysis, the results showed that the load did not affect the respective BIC in the adults (p = 0.177), but a decreased BIC was observed in the adolescents. Both the loaded and unloaded miniscrews showed significant differences between the adult group and the adolescent group in their respective BIC values (Fig. 3B).

### Haematoxylin-eosin staining and immunohistochemistry

**Fig. 4:**
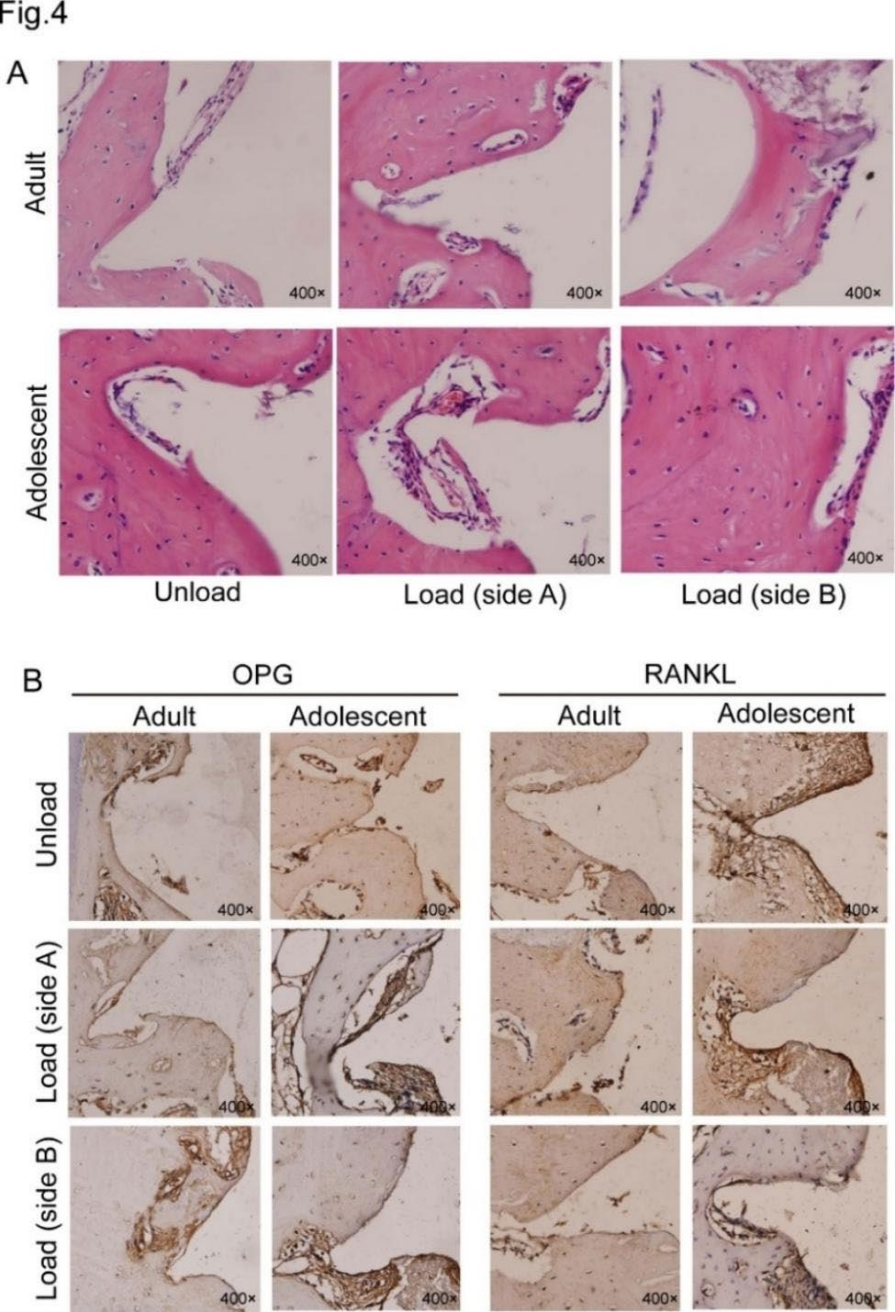
Haematoxylin-eosin staining and immunohistochemistry **A**. Haematoxylin-eosin staining of each group to observe osteogenesis and osteoclasis. **B**. The expression of OPG and RANKL protein in the peri-screw bone area by immunohistochemistry staining

The results of HE staining (Fig. 4A) revealed osteoblasts and osteoclasts in the peripheral bone of the miniscrews. Specifically, the osteoclasts of the juvenile beagles were stronger than those of the adult beagles in both the unloading and loading groups, which means that osteogenesis in juvenile beagles was better than that in adult beagles. The purpose of immunohistochemistry staining was to detect the expression of OPG and RANKL protein in the peri-screw bone area (Fig. 4B). The expression of OPG in bone around the miniscrews decreased after 10 weeks of force application in both groups compared with the unloading condition, and the reduction in side A of the applied miniscrews in the adolescent group decreased the most. The expression of RANKL was increased more in the adolescent group than in the adult group, and the increase was the largest on the A side of the miniscrews. The mean integrated optical density values are shown in Table S3. The ratio of RANKL/OPG expression increased in both groups; the increase on side A was the largest in the adolescent group, and the increase on side B was the smallest in the adolescent group. The results showed that the bone resorption on the A side of the adolescent miniscrews was the highest, and the bone resorption on the B side of the adolescent miniscrews was the lowest.

## Discussion

Considerable interest in miniscrews has grown because of the strong anchorage control they provide for executing hitherto impossible tooth movement[27]. However, loosening of miniscrews still occurs frequently in daily clinical practice due to the enormous number of patients, especially younger patients[13]. The present study was designed innovatively to further investigate the differences in anchorage stability of miniscrews influenced by age-related factors from the perspective of histology.

Orthodontic treatment in adolescents and adults displays multiple differences, such as duration, tooth movement parameters, accidental periodontal adverse effects, biological response and growth potential, which are of great concern for orthodontists[28–30]. There is a reduced rate of tooth movement in adults along with more pain and discomfort than in adolescents, and the discrepancy might be associated with the fact that the bone characteristics of adults differ from those of adolescents[31]. At the same time, the primary stability of miniscrews depends on bone characteristics and other factors such as implant geometric design (length, diameter, thread type, thread shape, thread pitch, thread design), and surgical technique-related factors (cortical predrilling insertion angle) [32–34]. Therefore, bone characteristics tend to be a prerequisite for differences in the outcomes of the two groups.

Some previous studies considered miniscrews to be an absolute anchorage, that could remain nearly immobile, or at least there was no clinically significant displacement compared with tooth movement [35, 36]. A growing number of studies have begun to focus on the displacement of miniscrews, and the displacement of the miniscrews were used to reflect the mechanical retention force [37]. Orthodontic loading-induced displacement distinguishes between primary and secondary displacement, it has been proposed that the sum of displacements should not exceed 1 mm[38]. In another study, the total displacement (mean value ≤ 0.78 mm) of forty-one miniscrews were measured submitted to force after a 5 months period[39]. These results are basically consistent with our study, the mean maximum displacement during 10 weeks was 0.625 mm observed in adolescent group.

Primary displacement refers to the immediate displacement subjected to functional load due to the elastic and plastic properties of the bones, and secondary displacement is defined as the long-term displacement of miniscrews due to bone remodelling processes[36]. There is a lower modulus of elasticity, bending strength, and bone density in bone of the younger, all of which contribute to the bone bending tendency under mechanical loading[26, 40, 41]. The initial retention of miniscrews also rely on mechanical locking provided by the adequate cortical bone thickness and bone mineral density [42, 43]. Data observed from a clinical point of view has suggested that the greater the cortical thickness, the lower will be the miniscrew displacement[38]. Studies have shown that adolescents presented with significantly thinner alveolar cortical bone thickness and lower cancellous bone density at different levels from the alveolar crest than adults[44, 45], implying that mechanical latches from the alveolar bone tend to be a weaker support in a growing population. This is one of the main reasons why the average displacement of miniscrews in the adult group was obviously shorter than that in the adolescent group, indicating the stability of miniscrew anchorage was compromised in adolescents with thinner cortical layers. After all, greater physiological anchorage loss also occurs in adolescents than in adults[46].

It has been demonstrated that a 1 mm zone of devitalized bone develops quickly around the miniscrews, particularly in the cortical region, due to surgical trauma and stress necrosis [47]. The devitalized interface cortical healing relies on continuous dynamic bone remodelling in cancellous bone to ensure lasting direct bone-to-miniscrew anchorage, which is associated with secondary displacement tightly. Accumulated researches have showed the influence of age on bone turnover marker and bone remodelling. Higher concentrations of bone turnover and metabolic indicators were observed in younger individuals[25], indicating that there would also be more secondary displacement in adolescent group. Besides, we implanted the miniscrews with an insertion angle ranging from 60° to 70° based on the previous suggestions[48], while a finite element analysis demonstrated that insertion of miniscrews at angles less than or greater than 90 degrees to the alveolar process bone might decrease the anchorage stability of the miniscrew[49]. The immediate compression force increased the stress concentration at cortical bone, the angular acceleration resulted in a composite motion combining more tipping movement at the initial stage. It is even more of a challenge for bone quality put forwards by the increased torque. Crucially, the stability is at the lowest level during the third week, just at the end of the resorptive phase, especially in sites with thin cortical layers[50, 51]. All the above mentioned is aligned with our results that the displacement of both groups tended to plateau in the first 3 weeks and decrease thereafter in the following weeks.

The bone dynamics adjacent to miniscrews are thought to be active to maintain a vital bone-implant interface, which is improved by both direct bone deposition and mineral tissue integration[52]. Histomorphometry is a useful technique for evaluating the MAR and cellular events. We employed the BIC and MAR as parameters for static and dynamic histomorphometric analysis, respectively. After implantation, not only mineralized bone tissue contact but also osteoblasts firmly attached to the miniature screws in the direct-contact area, with gaps of hundreds of microns in other areas where the recruiting osteogenic cells subsequently become stationary osteoblasts to secrete the osteoid matrix, followed by mineralization to form irregular woven bone that encroaches on the miniscrew surface[53, 54]. The BIC represents the amount of bone directly in contact with the miniscrew surface, and the MAR represents the rate of progression of the mineralization front between two consecutive fluorochromes as an index of osteoblast activity[55, 56]. An increase in the BIC and MAR would be desirable as the bone undergoes modelling and remodelling. In our study, the BIC of all miniscrews in adults and adolescents ranged from 32.37 to 66.85%, although both groups showed good osseointegration. It is still unclear whether a 60% BIC is sufficient and better for miniscrew stability and service than a 32% BIC; however, a higher BIC in adults better indicates the state of adaptation at the interface. Nevertheless, the MAR in adolescents was faster than that in adults. It is evident from the results that bone formative responses are higher in adolescents than in adults, while higher rates of osteoclast differentiation were also coupled with significantly increased bone turnover activity in the younger population[28]. That is why a faster MAR but lower BIC presents in adolescents, and as time goes on, their BIC would be similar owing to the faster MAR in adolescents than in adults. The higher bone turnover rate in the adolescent group was a double-edged sword, which complicated the achievement of optimal mechanical stability, leading to faster bone modelling and remodelling but weaker anchorage strength of miniscrews within a short time, and the stability of the miniscrews in adults seemed more reasonable.

In addition, the immune-expression patterns of RANKL and OPG also emphasized the difference between adolescents and adults. OPG is expressed by osteoblasts, which might prevent osteoclasis by binding to the key osteoclastogenic cytokine RANKL and is pro-osteoblastic in bone mineralization and calcium ion homeostasis[57]. The ratio of RANKL/OPG is a determinant in regulating the activation and function of osteoclasts[58]. Slower bone turnover progress as well as maintenance of the RANKL/OPG ratio were observed in adults, which further confirmed the anchorage strength results.

The major limitation of this research was the single design. We adopted one type of miniscrew, the same insertion method, force application, and placement sites to reduce other interference factors. Thus, it is difficult to illustrate and summarize the problems in all situations. As far as the present study is concerned, it is a pilot and exploratory study, we preliminarily excavated the anchorage strength and histomorphometric changes distinguishing the susceptibility to failure of orthodontic miniscrews between adolescent and adult groups.

## Conclusion

Miniscrews do not remain in the same position as skeletal anchors, and the amount of displacement was higher in adolescent group than that in adult group, reflecting the weaker anchorage strength of miniscrews in adolescents due to the higher bone turnover rate and active bone remodelling. Therefore, it is possible to apply orthodontic loading to the miniscrews in adult patients earlier, even immediately, but it is recommended to wait a period of time for the adolescents.

### Electronic supplementary material

Below is the link to the electronic supplementary material.

Supplementary Material 1

Supplementary Material 2

Supplementary Material 3

## Data Availability

The datasets used and/or analysed during the current study available from the corresponding author on reasonable request.
