# Supplementary material for: Comparative analysis of anchorage strength and histomorphometric changes after implantation of miniscrews in adults and adolescents: an experimental study in Beagles
Source: BMC Oral Health. 2023 Sep 5;23:639. doi: 10.1186/s12903-023-03318-y (PMC10478492; doi:10.1186/s12903-023-03318-y)
Supplement: Supplementary file 1 — Supplementary Material 1 [file 12903_2023_3318_MOESM1_ESM.pdf]

Table S1 Distance between two mini-screws with mechanical force (mm)

Animal	Load duration		
	0 w	3 w	10 w
1#	11.20; 11.52	11.15; 11.45	11.15; 11.40
2#	11.15; 10.98	10.90; 10.78	10.73; 10.65
3#	11.45; 10.85	11.28; 10.73	11.18; 10.65
4#	10.85; 11.35	10.70; 11.18	10.60; 11.05
5#	10.48; 11.08	10.25; 10.80	10.13; 10.68
6#	11.90; 11.75	11.63; 11.55	10.45; 11.43
7#	12.55; 11.60	12.25; 11.38	12.03; 11.18
8#	10.70; 11.15	10.08; 10.55	9.73; 10.28
9#	10.50; 11.30	10.15; 10.85	9.90; 10.58
10#	11.10; 10.75	10.70; 10.48	10.50; 10.25
11#	11.25; 11.15	10.88; 10.80	10.65; 10.55
12#	10.80; 10.98	10.48; 10.65	10.25; 10.43
