# Supplementary material for: Comparative analysis of anchorage strength and histomorphometric changes after implantation of miniscrews in adults and adolescents: an experimental study in Beagles
Source: BMC Oral Health. 2023 Sep 5;23:639. doi: 10.1186/s12903-023-03318-y (PMC10478492; doi:10.1186/s12903-023-03318-y)
Supplement: Supplementary file 2 — Supplementary Material 2 [file 12903_2023_3318_MOESM2_ESM.pdf]

Table S2 BIC of all the mini-screws（%）

Animals	Implantation site					
	P2-P3		P3-P4		P4-M1	M1
	Side A	Side B	Side A	Side B		
1	60.25	58.87	61.36	61.28	63.37	64.85
2	58.36	54.84	56.34	56.86	58.67	57.36
3	63.38	62.64	66.56	64.38	63.88	66.85
4	55.64	52.36	56.32	55.68	59.63	58.29
5	50.27	48.59	52.34	51.82	53.37	52.86
6	60.27	59.89	62.32	60.46	63.56	64.19
7	55.58	45.68	56.34	49.26	58.69	60.12
8	48.39	40.37	46.56	39.68	50.67	51.59
9	50.19	45.67	49.66	42.58	50.79	52.35
10	40.69	32.37	43.63	40.11	47.56	46.81
11	52.18	46.36	50.26	45.46	50.21	52.88
12	45.55	40.03	42.34	36.62	46.15	46.00
