# Supplementary material for: Comparative analysis of anchorage strength and histomorphometric changes after implantation of miniscrews in adults and adolescents: an experimental study in Beagles
Source: BMC Oral Health. 2023 Sep 5;23:639. doi: 10.1186/s12903-023-03318-y (PMC10478492; doi:10.1186/s12903-023-03318-y)
Supplement: Supplementary file 3 — Supplementary Material 3 [file 12903_2023_3318_MOESM3_ESM.pdf]

Table S3 Expression of RANKL/OPG in bone tissue around the mini-screws

Group		Unload	Load(side A)	Load(side B)
Adults	RANKL	0. 7429±0. 3213	1. 5026±0. 4856	1. 3628±0. 3769
	OPG	2. 6128±1. 6953	1. 8026±1. 7015	2. 0649±1. 5893
Adolescents	RANKL	1. 6624±0. 6032	3. 7546±1. 2689	1. 9846±0. 7298
	OPG	5. 3149±1. 7053	3. 0285±1. 3521	4. 3616±1. 5307
